# Supplementary material for: Modeling glioblastoma heterogeneity as a dynamic network of cell states
Source: Mol Syst Biol. 2021 Sep 16;17(9):e10105. doi: 10.15252/msb.202010105 (PMC8444284; doi:10.15252/msb.202010105)
Supplement: Supplementary file 6 — Source Data for Figure 5 [file MSB-17-e10105-s004.zip › Figure5A_sourcedata/GSEA_3017/hallmarks_stateF.GseaPreranked.1621934628066/HALLMARK_SPERMATOGENESIS.html]

Details for gene set HALLMARK\_SPERMATOGENESIS[GSEA]

|  || Dataset | state33017 |
| Phenotype | NoPhenotypeAvailable |
| Upregulated in class | na\_neg |
| GeneSet | HALLMARK\_SPERMATOGENESIS |
| Enrichment Score (ES) | -0.57313657 |
| Normalized Enrichment Score (NES) | -2.4163702 |
| Nominal p-value | 0.0 |
| FDR q-value | 0.0 |
| FWER p-Value | 0.0 |
Table: GSEA Results Summary

  

Fig 1: Enrichment plot: HALLMARK\_SPERMATOGENESIS      
 Profile of the Running ES Score & Positions of GeneSet Members on the Rank Ordered List

  

| PROBE | GENE SYMBOL | GENE\_TITLE | RANK IN GENE LIST | RANK METRIC SCORE | RUNNING ES | CORE ENRICHMENT || 1 | SCG3 |  |  | 130 | 0.698 | -0.0061 | No |
| 2 | HSPA4L |  |  | 215 | 0.579 | -0.0004 | No |
| 3 | GSTM3 |  |  | 348 | 0.488 | -0.0231 | No |
| 4 | MAP7 |  |  | 745 | 0.359 | -0.1736 | No |
| 5 | SLC12A2 |  |  | 782 | 0.351 | -0.1635 | No |
| 6 | ARL4A |  |  | 889 | 0.328 | -0.1865 | No |
| 7 | CNIH2 |  |  | 1135 | 0.287 | -0.2747 | No |
| 8 | PARP2 |  |  | 1665 | -0.305 | -0.4889 | No |
| 9 | TOPBP1 |  |  | 1781 | -0.354 | -0.5139 | No |
| 10 | NEK2 |  |  | 1815 | -0.371 | -0.5010 | No |
| 11 | RFC4 |  |  | 1887 | -0.413 | -0.5019 | No |
| 12 | TTK |  |  | 2047 | -0.533 | -0.5333 | Yes |
| 13 | NCAPH |  |  | 2104 | -0.652 | -0.5097 | Yes |
| 14 | AURKA |  |  | 2110 | -0.673 | -0.4617 | Yes |
| 15 | KIF2C |  |  | 2136 | -0.756 | -0.4164 | Yes |
| 16 | BUB1 |  |  | 2170 | -0.860 | -0.3670 | Yes |
| 17 | CDKN3 |  |  | 2199 | -0.979 | -0.3063 | Yes |
| 18 | CCNB2 |  |  | 2200 | -0.983 | -0.2329 | Yes |
| 19 | EZH2 |  |  | 2207 | -1.017 | -0.1596 | Yes |
| 20 | CDK1 |  |  | 2225 | -1.153 | -0.0811 | Yes |
| 21 | DBF4 |  |  | 2232 | -1.247 | 0.0094 | Yes |
Table: GSEA details [plain text format]

  

Fig 2: HALLMARK\_SPERMATOGENESIS: Random ES distribution      
 Gene set null distribution of ES for **HALLMARK\_SPERMATOGENESIS**
